# Transoral surgical approach to solitary fibrous tumors in buccal space with infratemporal tumor extension: A case report

**DOI:** 10.4317/jced.55736

**Published:** 2020-01-01

**Authors:** Andra Rizqiawan, Anindita Zahratur-Rasyida, Indra Mulyawan

**Affiliations:** 1 Department of Oral and Maxillofacial Surgery, Faculty of Dental Medicine, Universitas Airlangga

## Abstract

A solitary fibrous tumour (SFT) is a rare spindle-cell neoplasm of mesenchymal origin usually located in the pleura. It has been recently described as occurring in various head and neck sites, including the oral cavity. The purpose of this article is to report a case of SFT originating in the buccal space and extending into the infratemporal space treated by means of transoral approach surgery. A 25-year-old female patient reported to the Department of Oral and Maxillofacial Surgery, Universitas Airlangga Hospital, chiefly complaining of a painless lump in the left cheek which had been present for nine months. The diagnosis was arrived at on the basis of a combination of clinical investigation, imaging studies and histopathological examination (biopsy). The surgical approach involved transoral incision through the buccal mucosa. An SFT of buccal space may extend to nearby structures producing the anatomical challenge of removal through a transoral approach. Excisional biopsy involving a transoral approach is, nevertheless, considered appropriate because it produces an attractive aesthetic appearance, reduces morbidity from nerve/ vascular/ gland injury and promotes more effective healing.

** Key words:**Solitary fibrous tumor, buccal space, infratemporal space, transoral approach.

## Introduction

A solitary fibrous tumour (SFT) is an uncommon spindle-cell neoplasm of mesenchymal origin. An increasing incidence of the tumor has been found in the extrapleural sites, including the peritoneum, mediastinum, orbit, infratemporal fossa parapharyngeal space, upper airways and nose, salivary and thyroid glands and oral cavity. As documented in the literature, SFT accounts for 3% of all cases occurring in the oral region. When SFT develops in oral cavity it appears like a painless swelling, some of them may give rise to compression symptoms and with differential diagnosis lipomas, pleomorphic adenomas, schwannomas, fibrous histiocytomas, benign glandular tumors and dermoid cyst. Members of both sexes are equally affected, with patients being predominantly middle-aged or elderly. Symptoms depend on the site and depth of the tumour, but tend to be mostly asymptomatic slow growing masses and shows a high rate of positivity for CD34 on immunohistochemical staining (1-8).

The case reported here is one of an SFT in the left buccal space extending into the infratemporal region. The depth at which the tumor was located between the spaces rendered both distinguishing it from other soft tissue tumor and its removal challenging. An initial diagnosis was established through clinical examination, imaging and incisional biopsy. Definitive treatment ultimately consisted of excisional biopsy incorporating a transoral approach due to the benefits of an attractive aesthetic appearance, reduced morbidity from nerve, vascular and gland injury in addition to enhanced healing.

Case Report

**Figure 1:**
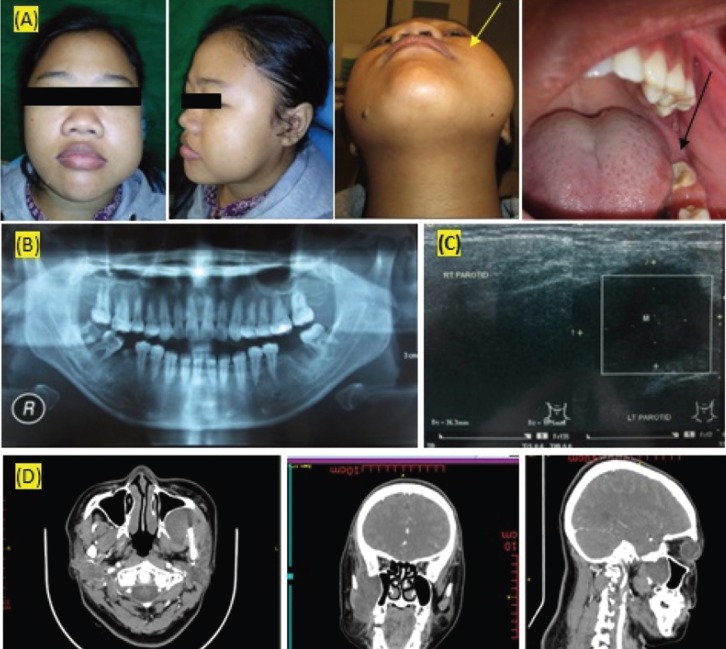
(A) Extra oral picture showed an asymmetry on the left cheek (black arrow) and (B) Orthopantomogram revealed no bone destruction of maxilla and mandible, (C) USG showed a hypoechoid solid mass on the left parotid area. (D) CT scan with contrast enhancement from coronal, axial, and sagital view revealed a slight enhancing soft tissue tumor with well border.

A 25-year old female reported to the Oral and Maxillofacial Surgery Department, Universitas Airlangga Hospital, Surabaya presenting the primary symptom of a painless lump in the left cheek she had first detected nine months earlier. The individual concerned did not complain of toothache or numbness and had no previous history of ill-health. Further oral examination revealed asymmetry of the left-hand side of the face without accompanying redness (Fig. 1A). Palpation indicated a firm, fully mobile, non-tender mass 4cms in diameter located relatively deep in the buccal space and extending into the left malar below the zygomatic arch. Palpation also confirmed the absence of lymphadenopathy in the head or neck region. Intraoral examination revealed a bulge in the left buccal mucosa, while palpation confirmed the presence of a well-defined, firm and non-tender mass located deep in the buccal space.

An initial orthopantomogram showed no destruction of the mandible and maxilla, but a post-extraction socket existed in the 36 region due to the patient having undergone a tooth extraction two months before (Fig. 1B). Ultrasonography (USG) of the left cheek indicated the presence of a discrete well-demarcated, hypoechoid solid mass2.6x2 cm in size located on or overlapping with the left parotid gland. There was no increase in internal in vascular flow or calcification of the mass (Fig. 1C).

A head and neck CT scan with contrast detected a developing soft tissue tumor 2.69x3.73x4.0 cm in size in the left buccal space (Fig. 1D) resulting in indentation and saucerization of the left posterior maxillary sinus wall, medial pterygoid, masseter and left temporal muscle which appeared as a slow growing tissue mass. The tumor originated in the buccal space and extended into the temporal space. The mass was fed by branches of the left buccal artery. The bone structure was normal.

A fine-needle aspiration biopsy was performed by puncturing an area from the extraoral to the intraoral site. Microscopic examination showed a hypocellular area consisting of a broad distribution of erythrocytes with a number of inflammatory cell histiocytes and neutrophils, but no signs of malignancy. An incisional biopsy was also performed through an intraoral incision on the left buccal which revealed tissue with wide fibroblast proliferation, extremely similar to a fibroma.

**Figure 2:**
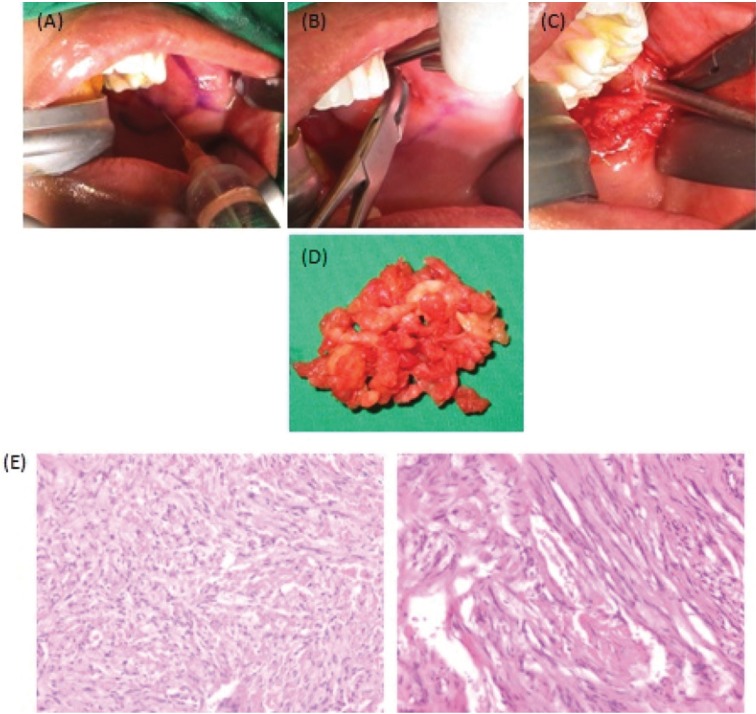
(A-C) Injection of vasoconstrictor 1:200.000 and Blunt dissection to reach the mass; (D) Tumor specimen excised from buccal space. (E) Microscopic view of the mass using HE staining with 20x and 40x magnification.

Following the securing of patient consent, an excisional biopsy using an transoral approach was performed under general anesthesia. First, the left buccal was palpated to localize the mass area which was subsequently marked with methylene blue. The incision site was also marked in the middle of the mass. Prior to incision, 1:200.000 of pehacain was injected as a vasoconstrictor to reduce oozing during surgery (Fig. 2A). A blunt dissection was performed post-incision as far as the mass area which was then completely separated (Fig. 2B,C). The tumor was capsulated but, being brittle, it tended to rupture during removal. Therefore, it proved necessary to conduct dissection in several steps in order to achieve total removal. Since the mass extended deep into the temporal space, the most difficult aspect of the removal came during dissection through the temporal space which was located immediately below the zygomatic arch.

The gross pathology of the tumor confirmed a total of five grams of specimen material (Fig. 2D). From the microscopic findings, it was evident that the tumor tissue was composed of fibroblast proliferation with spindle and flat nuclei arranged in a patternless structure within the collagen matrix. Partly dilatated vasculars were also present in the tumor resulting in its ultimate pathological diagnosis as a solid fibrous tumor (Fig. 2E).

No post-surgery complications of paraesthesia, paralysis or parotid injury were apparent and a routine follow-up conducted six months after the operation found no evidence of reoccurrence.

## Discussion

An SFT is a rare mesenchymal neoplasm generally considered to occur in ubiquitous interstitial stem cells situated within soft tissues. Although most appear in the parietal or visceral pleura or peritoneum, they can be present in other extrapleural sites, including: the mediastinum, lungs, liver, breasts, retroperitoneum, spine, meninges and extracranial head and neck regions. Symptoms depend on the site and depth of the tumour. Usually slow-growing and asymptomatic, it is described as having normal overlying skin and mucosa (1,2,9-11). The patient reported an unspecific slow-growing lump on her cheek which was not tender and had first appeared approximately nine months previously. Immunoreactivity for CD34, bcl-2 , CD-44, CD99 and Vimentin but is negative to keratin, EMA, S-100, desmin, smooth muscle actin and muscle specific actin was helpful to confirm the diagnosis of SFT (12,16).

**Figure 3:**
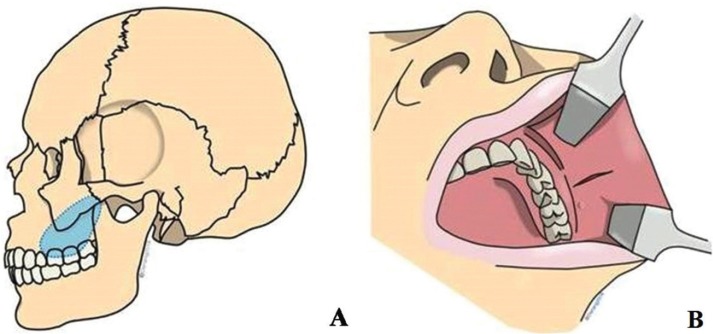
(A) Location of tumor showed in blue area, (B) Design of incision.

Although not pathognomonic, homogeneous or heterogeneous, attenuated enhancement is reported to be the most prominent feature of SFT revealed through CT and MR imaging. This characteristic is attributed to high vascularity because of the prominent vascular channels within the tumor. While remodeling of the adjacent bones may be observed in large, long-standing lesions, frank bone destruction represents an exceptional finding that should prompt suspicions of a malignant tumor (2,9). Extrapleural SFTs are almost always benign and cured by means of simple surgical excision (13). Stereotactic radiosurgery was treatment option in for recurrent solitary fibrous tumour and for patient refused surgery, percutaneous thermal ablation was proposed as a treatment alternative which can prevent surgical scar, reduced recovery time (14). The SFT affecting the patient was located in the buccal space, extended into the infratemporal space (Fig. 3A) and was treated with excisional biopsy using a transoral approach which was relatively rapid and straightforward with few complications.

The primary consideration regarding facial incisions is that of esthetics. The face is plainly visible to others and a conspicuous scar may constitute a cosmetic deformity potentially as troubling to the individual as the initial reason for the surgery being performed. The primary advantage of a transoral approach is that of concealing the intraoral scar. A second consideration for facial incision is the location of the muscles and nerves controlling facial expressions (N. VII) which can be traumatized if incision occurs in their proximity. This can result in facial paralysis which not only constitutes a severe cosmetic deformity but can, in addition, have significant functional ramifications (15). The other vital anatomical structure, the parotid gland, may also need to be taken into account. By adopting a transoral approach, such damage can be avoided.

Access to the mass was effected through an incision in the buccal mucosa (Fig. 3B), with care being exercised to avoid the parotid duct, before the fat pad and buccinators were penetrated and the oral cavity entered opposite the second molar (9,15). Since the mass extended as far as the temporal space, this method was more difficult than an extraoral approach which, in this case, could have used a Weber-Ferguson incision designed to avoid further complications, minimize and hide the scar and facilitate the complete removal of the SFT mass in order to prevent a recurrence of the condition. However, a transoral approach is usually considered first for a SFT located in the buccal space because patient satisfaction will increase due to the absence of a facial or extra-oral incision and the greater rate at which the mucosa heals compared to the skin.

Long-term follow up is mandatory for SFT patients because, in certain cases, clinical behavior does not correlate with histopathologic appearance. A slight but statistically significant increased risk of local disease recurrence was found in extrathoracic SFT (7-9).
